# Book Reviews

**Published:** 1905-02

**Authors:** 


### BOOK REVIEWS.

Gallstones and Their Surgical Treatment. By B. G. A. Moynihan, M.S. (Lond.), F.R.C.S., Senior Assistant Surgeon to Leeds General Infirmary, England. Octavo, 386 pages, illustrated, including nine colored insert plates. Philadelphia, New York, London: W. B. Saunders & Company. 1904. (Cloth, $4.00 net.)

The surgical treatment of gallstone disease is fast becoming the accepted method, not only in the presence of acute symptoms but upon assured diagnosis at any stage, or even in the absence of symptoms if gallstones be present. Mr. Moynihan is the apostle of the surgical treatment of gallstone disease and it is most appropriate that he has given us a treatise on the subject, because of his large experience and ripe judgment. The eleven chapters of his book are replete with interest and discuss every feature of the subject, from the anatomy of the gallbladder and ducts which forms the subject of the first chapter, to operations for obstruction of the common duct which are detailed in the final chapter.

One of the chief claims of this treatise to professional favor is its nature, being based upon the clinical experience of the author, which is very large. It will be remembered by many that Mr. Moynihan visited this country in May, 1903, and took part in the proceedings of the Congress of American Physicians and Surgeons at Washington. His discussion of the surgery of the stomach and of gallstone disease was such as to impress his audience with his knowledge and surgical skill.

The present work will do much to increase the confidence of the medical profession in the surgical treatment of the disease, particularly in the earlier stages of its manifestations during which, until comparatively recently, the practice has been to depend upon medicine instead of surgery,—upon drugs instead of the knife. Now, however, the most progressive physicians are ready to admit that, in the presence of obstructive gallstone disease with urgent symptoms, they are powerless to relieve, and the aid of the surgeon must be sought in order to save life.

We cannot speak too highly of Mr. Moynihan's modest presentation of this great subject and we hope his book will make its way into the hands of every physician as well as surgeon, who makes any pretense to keep pace with the progress of professional events.

---

Diseases of the Liver, Gallbladder, and Bile Ducts. By H. D. Rolleston, M.D., Physician to Saint George's Hospital, London. Octavo, 794 pages, illustrated, including seven colored insert plates. Philadelphia, New York, London: W. B. Saunders & Company. 1904. (Cloth, $6.00 net.)

Great importance of late has become attached to the diseases of which this book treats, particularly because many of them are amenable now to surgical intervention. Without doubt this is the most comprehensive treatise relating to diseases of the liver and its appendages that has appeared in English, and is destined to become recognised authority by teachers and practitioners of medicine.

The recent acquisition of island possessions in the tropics by the United States has stimulated the study of diseases incident to the climate on the part of American physicians. This treatise will assist very much in the acquirement of knowledge concerning many tropical diseases. In this relation the section on tropical abscess of the liver has special interest and significance. That the disease is closely related, if not largely dependent upon, dysentery, is now believed; indeed, it has been so demonstrated to the satisfaction of the most careful observers of tropical diseases. Such being the case intestinal disease, and especially dysentery, possesses increased importance for the student of tropical diseases. Of course, other causes of tropical abscess apart from dysentery are to be considered, but dysentery seems to be allotted chief place at present.

Probably no disease of the liver has received more attention or been the subject of greater controversy than cirrhosis of that organ, primary or secondary. Rolleston assigns over one hundred and sixty pages to its consideration, and it may be remarked that nowhere has the subject been discussed so clearly, so completely, and we might add, so masterfully as here. Syphilitic disease of the liver is only second in importance to the foregoing because it is less frequent than cirrhosis. But Rolleston gives a complete clinical picture of the disease as well as a description of its several methods of manifestation and a terse account of the gummata.

Gallstone disease is the subject in the final section of the book, and the entire volume is filled with material that can only be appreciated by studying it carefully from beginning to end.

---

Lectures to General Practitioners on the Diseases of the Stomach and Intestines. By Boardman Reed, M.D., Professor of Diseases of the Gastrointestinal Tract, Temple College, Philadelphia. Octavo, 1021 pages. Illustrated. New York: E. B. Treat & Company. 1904. (Price, $5.00.)

A book of lectures of diseases of the stomach is to be welcomed at this time because, among other reasons, a treatise in such form is apt to bring out the latest and best methods of diagnosis and treatment and, moreover, to present them in readable, not to say entertaining form. This is precisely what this author has accomplished. While it is apparent that he has not made startling discoveries nor demonstrated great and original methods he has, nevertheless, done the profession of medicine some service in giving it a good treatise constructed on modern pathology, etiology, diagnosis and treatment. It is also quite apparent that the incongruities of style as well as defective grammatical constructions here and there are incident probably to the lecture method without careful revision, but these will disappear no doubt when the next edition is put forth.

We confess it is difficult to understand why an author who has made a modern book, medically speaking, should mar it by permitting it to print violations of present day orthography. The spelling of words differs under varying conditions of time and environment. The orthography of today is the result of evolution, differing materially from that of the eighteenth century, as will the orthography of the twenty-first century differ from ours. The medical profession is slow to adopt changes, but there is no good reason why it should not be consistent. If "hemorrhage," why "anæmia," "ætiology," and the like. Another thing--in expressing the possessive of a proper name ending in *s*, an apostrophe and an *s* should be added: thus, Boas's, instead of Boas'. If we, as a people, are not ready to adopt the so-called reform spelling, we may at least use simple English, and "write as we speak."

If a medical student in the pursuit of his studies relating to diseases of the stomach and intestines should buy this book and study it closely, he will prepare himself to deal with those questions intelligently, and will equip himself to meet all examinations, relating to them.

---

A Textbook of Clinical Diagnosis by Laboratory Methods. For the use of Students, Practitioners, and Laboratory Workers. By L. Napoleon Boston, A.M., M.D., Associate in Medicine and Director of the Clinical Laboratories of the Medico-Chirurgical College, Philadelphia. Octavo, 547 pages, with 320 illustrations, many in colors. Philadelphia, New York, London: W. B. Saunders & Company. 1904. (Cloth, $4.00 net; sheep or half morocco, $5.00 net.)

The laboratory is playing such important part in the diagnosis of most diseases that it is almost as difficult to keep pace with its ever improving methods, as to follow the advances of all the other diagnostic processes. The author designs this book as an aid to the medical student and general practitioner, having adapted it in particular to their needs, by limiting its scope to such simple technic as can be carried out without complicated or elaborate armament. The blood, as might be anticipated, receives first attention and its study is given in minute detail, including the recent methods of examination and staining, which are admirably illustrated.

Next to the blood, the urine claims attention, each, however, covering about the same space,—one hundred and forty pages,—and each receiving the most exhaustive description, though in neither section is there any display of overdoing, all being told in simple words that are not used without necessity or meaning; that is to say, every word seems to have been weighed and none is used that has not its precise place.

The section on the urine will prove of interest to every practising physician, because he must needs know all that can be learned by subjecting this excretion to the most careful scrutiny; and in this work there is so much that is practical and not a little that is original in the studies of the urine. All the newer methods of investigation are described and the significance of the findings are enumerated. The studies of animal and vegetable parasites possess peculiar interest at this time when their significance is being interpreted with more accuracy than heretofore, and when new ones are being found with sufficient frequency to lend interest constantly to the subject.

The illustrations are numerous and creditable,—many being of unusual excellence, especially those in colors delineating the blood under various conditions. It is a book that is likely to become a part of the textbook equipment of every student who wishes to master the art of clinical diagnosis.

---

A Treatise on Obstetrics. For Students and Practitioners. By Edward P. Davis, A.M., M.D., Professor of Obstetrics in Jefferson Medical College, Philadelphia. Second edition, thoroughly revised and enlarged. Octavo, 800 pages, with 274 engravings and 39 full-page plates in colors and monochrome. New York and Philadelphia: Lea Brothers & Company. 1904. (Cloth, $5.00 net; leather, $6.00 net.)

Something like eight years ago (February, 1897,) the Journal had the pleasure of reviewing the first edition of this treatise, pointing out in considerable detail its many excellent features. Since that time many advances have been made in obstetrics, hence a new edition intended to embrace all those of value is sent out.

One of the first characteristics of the new edition that attract attention is its increase in size over the old, the new containing 809 pages as against 553 pages in the old, and this difference of 256 pages is a good expression of the relative advancement of the science and art of obstetrics beyond its status when the book was issued the first time. A similar note may be made of the illustrations for, whereas the first book contained 217 engravings and 30 plates, this one presents 274 engravings and 39 plates.

The author is a teacher of experience and is capable of giving expression in writing to that which is most needful to him who is in search of light, be he undergraduate pupil or general practitioner, junior or senior; or, again, if he happens to be a teacher of obstetrics,—even all these may profit by studying or examining this treatise.

The methods of treatment advised by the author are based on his personal experiences. Without entering into a detailed account of the author's methods, we may, nevertheless, reaffirm our opinion as previously expressed,—that it is a popular textbook and takes rank among the best of obstetric treatises. Besides giving full information from the beginning to the end on the subject of obstetrics proper, it deals also with the infant in health, and in disease, and finally, with the jurisprudence of obstetrics. The latter topic is rarely taken up in such a treatise, and this fact alone increases the value of the book to junior physicians.

---

The Practical Application of the Roentgen Rays in Therapeutics and Diagnosis. By William Allen Pusey, A.M., M.D., Professor of Dermatology in the University of Illinois; and Eugene W. Caldwell, B.S., Director of the Edward N. Gibbs Memorial *x*-ray Laboratory of the University and Bellevue Hospital Medical College, New York. Second edition, revised and enlarged. Octavo, 690 pages, with 195 illustrations, including four colored plates. Philadelphia, New York, London: W. B. Saunders & Company. 1904. (Cloth, $5.00 net; sheep or half morocco, $6.00 net.)

The interest manifested in the mysterious realm of electrical medicine reaches the extreme of curiosity when two editions of a book on *x*-ray diagnosis and treatment are issued within a single year. The appetite for the spectacular is very great, not alone among patients but it must be admitted that physicians, likewise, are affected by the same inordinate hunger, perhaps in more moderate degree let it be said to professional credit. There is danger, however, that even physicians will become afflicted with acute indigestion if the morbid appetite for electrical display is not checked in some manner.

In September of last year we spoke in praise of Pusey and Caldwell's work in the *x*-ray field. We expressed the opinion that the influence of their book would do much to lift the practice with *x*-rays up to a higher level and to disarm the fakir of much of his stock in trade. The early demand for a second edition indicates that their work has taken root, not only in the direction named, but that it has appealed to students of electricity in general as well as to surgeons and dermatologists in particular.

So little new has developed in this direction of late, the authors in presenting a new edition have done little more than verify some of the clinical data previously given out. During 1903, many wrote, while, but few said anything that would add to the common stock of knowledge on this topic.

This book is printed on heavy calendered paper,—a detriment we think; it makes the book heavy—though, of course, its pages look fine. It is illustrated much as before, and is a handsome volume.

---

Diet in Health and Disease. By Julius Friedenwald, M.D., Clinical Professor of Diseases of the Stomach in the College of Physicians and Surgeons, Baltimore; and John Ruhräh, M.D., Clinical Professor of Diseases of Children. Octavo, 689 pages. Philadelphia, New York, London: W. B. Saunders & Company. 1904. (Cloth, $4.00 net.)

The first attempt to cover a distinct field in medical literature is always marked by difficulties and embarrassments to be overcome, that require special qualifications of mind and training to deal with. This book contains material collected from all available sources brought together for the first time in readable form, and will prove of value to every physician who treats disease. The regulation of diet is one of the first duties of the medical man nowadays, and he needs at hand just this work to aid his judgment or assist his memory.

The work has been constructed with special reference to the necessities of the bedside and the consulting room,—of the clinician, hospital assistant or resident physician, general physician, and student of medicine. It is adapted to the sick and the well, to the invalid and the infant, to the over-fed and under-nourished. Here, too, are diet lists for the nurse to study and become familiar with, and for the physician to utilise; rectal feeding is fully considered and food adulteration is amply dealt with, and there are many other features which contribute to make this book the most useful of its kind yet published.

A Textbook of Physiological Chemistry. For Students and Practitioners of Medicine. By Charles E. Simon, M.D., author of Simon's Clinical Diagnosis. Second edition. Revised and enlarged. Octavo, 500 pages. Philadelphia and New York: Lea Brothers & Company. 1904. (Cloth, $3.25 net.)

The original edition of this useful textbook was issued about three years ago. It proved most helpful to medical students and physicians who had not been able to study adequately the subject of physiological chemistry.

The author discusses in detail the three great classes of food stuffs,—the albumins, the carbohydrates, and the fats,—with which every medical student and physician should familiarise himself. The chemistry of digestion is set forth in a full and interesting manner; the excretions are given important heed; reconstructives are amply considered, and the glandular organs receive due attention; in short, there is no omission in this book that can be pointed out, which has any important bearing on the subject. It has been revised with great care, such additions having been made as were necessary to meet changed conditions, including an appendix of laboratory exercises, and the whole treatise has been brought down to the immediate present, as far as it is possible with ever changing knowledge on many of the questions involved in these researches.

It is a work of today in every respect and exemplifies the importance of a familiarity with laboratory studies on the part of the student of medicine; nowhere else can he obtain that knowledge which is so well imparted in this book, and with which he may enter the laboratory prepared to assist in working out new problems.

---

The Surgical Treatment of Bright's Disease. By George M. Edebohls, M.D., Professor of the Diseases of Women in the New York Post-Graduate Medical School and Hospital. Octavo, 327 pages. New York: Frank F. Lisiecki, Publisher. 1904.

The distinguished author of this monograph is a pioneer in the field which his book contemplates, his method having been first proposed by him in an article published in the *Medical News,* April 22, 1899, and which constitutes the opening chapter in this work. The book, in its first two-fifths, consists of a collection of essays pertaining to the surgical treatment of Bright's disease, published by the author at various times and in different periodicals during the past five years. The remaining three-fifths consisting of new material, deals mainly with the results of the method advocated and put in practice by Edebohls. The whole subject is hardly more than sub judice at the present time, yet what is here said in relation to it possesses the keenest interest for both physicians and surgeons.

---

Textbook of Nervous Diseases and Psychiatry. By Charles L. Dana, M.D., Professor of Nervous Diseases and (ad interim) of Mental Diseases in Cornell University Medical College. Sixth revised and enlarged edition. Octavo, 690 pages. Illustrated by 244 engravings and three plates in black and colors. New York: William Wood & Company. 1904. (Price, $4.00.)

This author is among the leading teachers and writers on the subjects dealt with in this treatise. He has written one of the best books in English, relating to nervous and mental diseases. This is its sixth edition within a comparatively few years. It is adapted to the use of specialists, and those who desire to pursue inquiries relating to the topics with which it has to do; those, we mean, who already possess some knowledge of them and desire more; those who are seeking, earnestly and studiously, the best there is, for here they will find facts and thoughts expressed tersely and clearly, that will lead them to a better understanding of these often subtle and sometimes incomprehensive diseases.

When a book has advanced to its sixth edition so promptly, and when, also, it has been more than once copiously reviewed in these columns, it would seem that it is quite unnecessary to say more at this time.

Dana announces that he is in sympathy with the proposition that makes as few people insane as possible, of narrowing the conception of the major psychoses and enlarging that of the minor,—views that will appeal to everybody who has studied nervous and mental disease to any purpose.

---

Kirke's Handbook of Physiology. Revised by Frederick C. Busch, B.S. M.D., Professor of Physiology, Medical Department, University of Buffalo. Fifth American revision, with 535 illustrations, including many in colors. New York: William Wood & Company. 1904.

The chief interest in this edition of this standard work on physiology resides in the fact that it has been revised by a Buffalo physiologist, who is steadily rising to the first rank among the younger teachers of this topic. Dr. Busch has performed his work well, having made only those changes deemed absolutely necessary to keep the book up to the level of present methods. The blood and its circulation necessarily come in for a share in the revision, the studies in this field of late having led to a modification of former teachings, especially regarding blood pressure and its influence on the economy in health and disease. Other subjects in which changes became necessary to meet advancing knowledge were respiration, digestion, and muscle-nerve physiology. Interesting studies in muscle contractility are detailed in chapter fourteen. Some changes have been made in the illustrations, both by adding new and eliminating old ones, in order to more clearly explain the text.

In its present state and under its auspicious rehabilitation Kirke's handbook is likely to occupy the front row on the physiological stage for some time to come.

---

A Textbook of Materia Medica, including laboratory exercises in the Histologic and Chemic Examinations of Drugs. For Pharmaceutic and Medical Schools, and for Home Study. By Robert A. Hatcher, Ph.G., M.D., Instructor in Pharmacology in Cornell University Medical School of New York City; and Torald Sollmann, M.D., Assistant Professor in Pharmacology and Materia Medica in the Medical Department of the Western Reserve University of Cleveland. Duodecimo, 410 pages, illustrated. Philadelphia, New York, London: W. B. Saunders & Company. 1904. (Flexible leather, $2.00 net.)

It is a good plan to make the study of materia medica as attractive as possible by creating books and methods that please the eye and engage the mind. This textbook is constructed on the lines mentioned, by adopting modern methods in the teaching and study of the subject; in other words, by popularising the laboratory plan of teaching and by insisting upon objective as against didactic instruction. In this way much of the dryness hitherto attached to materia medica is eliminated and a dull topic is changed to one of interest. It became necessary for the authors to create a book which would give the necessary information in convenient compass in order to impart instruction by this method inasmuch as none such was in existence; hence this work, which is commended for its compact, systematic, and scientific presentation of the laboratory method as applied to materia medica.

---

A Manual of Experimental Physiology. By Winfield S. Hall, Ph.D., M.D., Professor of Physiology, Northwestern University Medical School, Chicago. Octavo, 245 pages. Illustrated. New York and Philadelphia: Lea Brothers & Company. 1904. (Price, $2.75.)

An experienced teacher presents in this attractive volume the fruits of his labors in a most important field of experimental research.

A good impression of the scope of the volume may be formed from the following summary of its contents. After an introduction to the subject, part I. on experimental general physiology, takes up cytology and the general physiology of muscles and nerve tissue. In part II, on special physiology, will be found the chapters on the circulation of the blood; on respiration; normal hematology; digestion and absorption; vision; the nervous system and the muscular system.

An important feature of the work is the attention given to the physiological action of drugs,—a matter of extreme value. Too little attention, we fear, is paid to this subject by the average physician, not to speak of its neglect by the undergraduate. We know of no more useful preparation for medical practice than the mastery of the teachings of this manual.

---

Saunders' Question Compends: Essential of Chemistry, Organic and Inorganic. Containing also questions on Medical Physics, Chemical Philosophy, Medical Processes, Toxicology, etc. By Lawrence Wolff, M.D., formerly Demonstrator of Chemistry at the Jefferson Medical College, Philadelphia. Sixth edition, thoroughly revised. By A. Ferree Witmer, Ph.G., formerly Assistant Demonstrator in Physiology at the University of Pennsylvania. Duodecimo, 225 pages, illustrated. Philadelphia, New York, London: W. B. Saunders & Company. 1904. (Cloth, $1.00 net.)

With so full a title page as the foregoing little need be said of a sixth edition of this compend, excepting to remark that it now contains material on organic chemistry, organotherapy, and the substituted ammonias, besides titles on the recent discoveries in physics, and inorganic chemistry. The book deals with a topic that peculiarly fits a compend, and this compend particularly fits the topic with which it deals.

A Textbook of Diseases of Women. By Charles B. Penrose, M.D., formerly Professor of Gynecology in the University of Pennsylvania. Fifth edition, thoroughly revised. Octavo volume of 539 pages, with 221 illustrations. Philadelphia, New York, London: W. B. Saunders & Company. 1904. (Cloth, $3.75 net.)

Two years ago the fourth edition of this treatise was noticed in these columns, and now comes a fifth,—a remarkable showing of popularity. The author, who was formerly professor of gynecology at the University of Pennsylvania, is one of the successful younger gynecologists and appears to know what the student requires in the pursuit of this branch of medical science.

The present edition shows extensive revision and it has not only been reprinted but recopyrighted, so that it brings the material down to the present moment, as far as its practical teachings are concerned. It is essentially a book for students of medicine and appears to be appreciated by them from the remarkable demands made for it.

---

How to Cook for the Sick and Convalescent. Arranged for the Physician, Trained Nurse, and House Use. By Helena V. Sachse. Second edition, revised and enlarged. Philadelphia: J. B. Lippincott Company. 1904.

It is an art to cook properly for well persons; it is a science to cook for the sick. Frequently the prompt recovery of a sick person depends quite as much upon the cook as the doctor. In this book one may learn not only the way to make food toothsome, but also how to make it serve the highest purposes of nutrition without taxing digestion unduly, or delaying the assimilative processes. Miss Sachse has called to her aid what might be called, with great propriety, the chemistry of cookery, and has produced one of the most helpful adjuncts of the armamentarium of the sick room that has come within our purview.

---

A Handbook of Surgery. For Students and Practitioners. By Frederic R. Griffith, M.D., Surgeon to the Bellevue Dispensary, New York City. Duodecimo volume of 579 pages, containing 417 illustrations. Philadelphia, New York, London: W. B. Saunders & Company. 1904. (Flexible leather, $2.00 net.)

It is well, since there must be handbooks, to have as much completeness as possible in the presentation topics, especially when surgery is the subject. This book is encyclopedic in its method of dealing with details, and is made to embrace not only general but special surgery, including many items not surgical strictly speaking, yet of value to those who practise surgery. It is illustrated with wood cuts and is printed and bound in the same general style as Stevens's Manual of Medicine, to which we presume it is intended to be a companion. In any event, this is a form of handbook that will be ever popular with undergraduates preparing for examination, and with junior physicians looking for hints.

REGIONAL MINOR SURGERY. By GEORGE GRAY VAN SCHAICK, M.D., Consulting Surgeon to French Hospital, New York. Second edition, enlarged and revised, 228 pages. Illustrated. New York: International Journal of Surgery Company. 1904. (Price, $1.50.)

A book of this kind in the hands of the beginner in surgical practice should do much good. It serves as a reminder in many conditions where deftness and a knowledge of technic may prevent formidable secondary complications that might arise from neglect or delay, and which, then, in all probability would require major surgical procedures.

The author in this second edition has made revisions and added some material, which brings it forward to the present time, and makes it a useful book for the general practitioner of medicine to have at hand.

---

UNIVERSITY OF PENNSYLVANIA. Contributions from the William Pepper Laboratory of Clinical Medicine. (Reprints). No. 4. Philadelphia. 1903.

These several reprints bound together in this form become convenient for reference, but their value would be much increased if a good index were added. The topics dealt with in this number includes diseases of the nervous system, injury of the cord and fractures of the vertebræ, hydrophobia, feeding by the rectum, percentage modification of milk for infants, acetanilid poisoning, diseases of the eyes, blood pressure, dermoid ovarian cyst, carcinoma of the appendix, and several other papers. The aggregation represents much excellent work in the laboratory that issues the book.

